# Hesperetin Nanocrystals Improve Mitochondrial Function in a Cell Model of Early Alzheimer Disease

**DOI:** 10.3390/antiox10071003

**Published:** 2021-06-23

**Authors:** Lukas Babylon, Rekha Grewal, Pascal-L. Stahr, Ralph W. Eckert, Cornelia M. Keck, Gunter P. Eckert

**Affiliations:** 1 Biomedical Research Center Seltersberg (BFS), Laboratory for Nutrition in Prevention and Therapy, Institute of Nutritional Sciences, Justus Liebig University, Schubertstr. 81, 35392 Giessen, Germany; lukas.babylon@ernaehrung.uni-giessen.de (L.B.); Rekha.Grewal@ernaehrung.uni-giessen.de (R.G.); 2 Department of Pharmaceutics and Biopharmaceutics, Philipps Universität, Robert-Koch-Str. 4, 35037 Marburg, Germany; pascal.stahr@pharmazie.uni-marburg.de (P.-L.S.); ralph-walter.eckert@pharmazie.uni-marburg.de (R.W.E.); cornelia.keck@pharmazie.uni-marburg.de (C.M.K.)

**Keywords:** Alzheimer disease, mitochondria, ROS, mitochondria dysfunction, nanoparticles, hesperetin, amyloid beta, peroxidase activity

## Abstract

Mitochondrial dysfunction represents a hallmark of both brain aging and age-related neurodegenerative disorders including Alzheimer disease (AD). AD-related mitochondrial dysfunction is characterized by an impaired electron transport chain (ETC), subsequent decreased adenosine triphoshpate (ATP) levels, and elevated generation of reactive oxygen species (ROS). The bioactive citrus flavanone hesperetin (Hst) is known to modulate inflammatory response, to function as an antioxidant, and to provide neuroprotective properties. The efficacy in improving mitochondrial dysfunction of Hst nanocrystals (HstN) with increased bioavailability has not yet been investigated. Human SH-SY5Y cells harboring neuronal amyloid precursor protein (APP_695_) acted as a model for the initial phase of AD. MOCK-transfected cells served as controls. The energetic metabolite ATP was determined using a luciferase-catalyzed bioluminescence assay. The activity of mitochondrial respiration chain complexes was assessed by high-resolution respirometry using a Clarke electrode. Expression levels of mitochondrial respiratory chain complex genes were determined using quantitative real-time polymerase chain reaction (qRT-PCR). The levels of amyloid β-protein (Aβ_1-40_) were measured using homogeneous time-resolved fluorescence (HTRF). ROS levels, peroxidase activity, and cytochrome c activity were determined using a fluorescence assay. Compared to pure Hst dissolved in ethanol (HstP), SH-SY5Y-APP_695_ cells incubated with HstN resulted in significantly reduced mitochondrial dysfunction: ATP levels and respiratory chain complex activity significantly increased. Gene expression levels of RCC I, IV, and V were significantly upregulated. In comparison, the effects of HstN on SY5Y-MOCK control cells were relatively small. Pure Hst dissolved in ethanol (HstP) had almost no effect on both cell lines. Neither HstN nor HstP led to significant changes in Aβ_1-40_ levels. HstN and HstP were both shown to lower peroxidase activity significantly. Furthermore, HstN significantly reduced cytochrome c activity, whereas HstP had a significant effect on reducing ROS in SH-SY5Y-APP_695_ cells. Thus, it seems that the mechanisms involved may not be linked to altered Aβ production. Nanoflavonoids such as HstN have the potential to prevent mitochondria against dysfunction. Compared to its pure form, HstN showed a greater effect in combatting mitochondrial dysfunction. Further studies should evaluate whether HstN protects against age-related mitochondrial dysfunction and thus may contribute to late-onset AD.

## 1. Introduction

To date, more than 50 million people have developed Alzheimer disease (AD), and by the end of 2050, over 152 million will be affected [1]. AD is the most common form of dementia and has its origin in various disorders of the brain [2]. Despite intensive research, AD is not curable yet. It is only possible to treat symptoms, which can slow, but not stop, progression of the disease. One possible trigger of AD is overexpression of amyloid β-protein (Aβ). An imbalance in production and removal leads to accumulation and aggregation in the brain, resulting in inflammatory reactions, reactive oxygen species (ROS) production, and the loss of neurons, which lead to dementia [3]. However, clinical data on the efficacy of drugs that target Aβ are inconsistent with the Aβ hypothesis, and its general rejection is currently debated [4,5,6]. The multifactorial pathology of AD makes it difficult to develop viable therapies, and research should focus on novel targets. One promising target is mitochondrial dysfunction, which represents a final common pathway of brain aging and dementia [7].

Reports suggest that mitochondrial dysfunction is an early event in the development of AD [8,9]. Almost all mitochondrial functions are impaired in AD [7,10]. The first signs of mitochondrial dysfunction are reduced glucose consumption [11] and reduced activity of key enzymes in the oxidative metabolism [12,13]. The limited function of the electron transport chain (ETC) is due to decreased activity in complexes I and IV. This results in decreased membrane potential and ATP production [14,15]. These defects, in turn, lead to increased oxidative stress, which has a further negative effect on mitochondrial function [16].

One possible approach to prevent mitochondrial dysfunction is the use of polyphenols, especially flavonoids [17]. One of the flavonoids, Hst, is the aglycon of hesperedin, a major flavonoid in orange juice but also found in high amounts in the peel of oranges and other citrus fruits in [18,19]. HstP, which was first extracted from hesperidin by hydrolysis in 1928 [20], is a bioactive molecule that can act in the body in many different ways. In mice, HstP has a lipid-lowering effect by decreasing cholesterol and triacylglycerides, and it has a positive effect on mouse hearts, improving blood pressure and protecting against fibrosis [21,22]. That HstP might not affect the lipid profile and blood pressure in randomized clinical trials is probably due to its quite low bioavailability, which is 15% [23,24]. Various strategies are available to overcome poor solubility, and drug nanocrystals represent one of the most powerful formulation strategies to enhance the kinetic solubility and dissolution rate of poorly soluble drugs.

Hst is a natural flavonoid with high antioxidant activity, poor water solubility, and lipophilic characteristics [25]. Even though liposomes are rather crude biomimetic models of biological cell membranes, data have shown that liposome electrokinetic chromatography (LEKC) can be used to predict the behavior of compounds in living systems [26]. For hesperetin, studies have shown high experimentally determined distribution constants (log KD) of about 2.25–3.65. They claim that hesperetin belongs to the category of antioxidants that preferentially interact with hydrophobic phospholipid membrane [26]. It has been shown in an in vitro model that Hst may cross the blood–brain barrier [27]. Hst was also reported to act as a radical scavenger [28].

We recently reported high antioxidative capacity of Hst nanocrystals using a DPPH assay [25]. In a study using PC12 cells, HstP provided protection against H_2_O_2_-induced oxidative stress. Incubation with HstP reduced cell viability, protected against membrane destruction, intercepted ROS formation, increased catalase activity, and protected against H_2_O_2_-induced reduction in mitochondrial membrane potential [29]. Nanoparticles are smaller than 1000 nm, but larger than 100 nm [30]. Nano-sized formulations are particularly suitable for substances that are difficult to dissolve. Furthermore, the size of these newly formed compounds can increase the bioavailability of otherwise poorly available substances. A promising approach could be using nano-hesperetin (HstN) to improve the low bioavailability. In a study employing rats injected intracerebroventricularly with streptozotocin, HstN improved their memory and recognition. In addition, the antioxidative enzyme activity and glutathione levels were increased in contrast to HstP [31]. At the same time, the nano-form protects against premature degradation of the substance and thus prolongs its stay in the bloodstream [32,33]. In this study, we examined the effects of Hst in both nano and pure form on mitochondrial function in SH-SY5Y-APP_695_ cells, an in vitro model for the initial phase of AD [34,35].

## 2. Materials and Methods

### 2.1. Hesperetin Nanocrystals

Hst was purchased from Exquim S.A. (Spain). Alkyl polyglycosid (Plantacare 2000 UP^®^; PC) was used as a stabilizer. Production of hesperetin nanocrystals (HstN) was achieved by small-scale milling modified after Romeo et al. [30]. For this, coarse suspension was filled in a 2 mL glass vial with 3 stirring bars and yttria-stabilized zircon oxide milling beads (diameter 1 mm; Retsch, Haan, Germany) with a suspension-to-bead ratio of 1:1 (*v*/*v*). All vials were stirred on a magnetic stirring plate (IKA RCT standard, Haan, Germany) at 1200 rpm in ice water for 8 h. After production and before treatment of cells, particle size was strictly monitored. The average particle size was determined by photon correlation spectroscopy using a ZetaSizer NanoZS (Malvern-Panalytical, Kassel, Germany), laser diffraction using a Mastersizer 3000 (Malvern-Panalytical, Kassel, Germany), and light microscopy using an Olympus BX53 light microscope (Olympus Cooperation, Tokyo, Japan) equipped with an Olympus SC50 CMOS color camera (Olympus Soft Imaging Solutions GmbH, Nord, Germany) according to [25]. Since polyphenols have the potential to act as pan-assay interference compounds [36], we used PAINS-Remover at https://www.cbligand.org/PAINS/ (accessed on 25 May 2021) to check whether hesperetin would be likely to interfere with screening technologies [37]. We found no evidence that Hst would act as such a compound.

### 2.2. Cell Culture

Human neuroblastoma SH-SY5Y cells were cultured at 37 °C under an atmosphere of 5% CO_2_ in DMEM supplemented with 10% (*v*/*v*) heat-inactivated fetal calf serum, 60 µg/mL streptomycin, 60 units/mL penicillin, 0.3 mg/mL hygromycin, MEM non-essential amino acids, and 1 mM sodium pyruvate 1%. SH-SY5Y cells were stably transfected with DNA constructs harboring human wild-type APP695 (APP) or its empty expression vector pCEP4 (Invitrogen, Europe) alone as control (MOCK); for details; please refer to [35]. Cells were passaged every 3 days and were used for experiments when they reached 70–80% confluence.

### 2.3. Cell Treatment

Cells were incubated with concentrations of 0.01 to 10 µM HstN or HstP for 24 h after they reached confluence of 80%. Ethanol (1%) was used as a control for HstP and PlantaCare (PC) for HstN.

### 2.4. Homogeneous Membrane Integrity Measurement

A homogeneous membrane integrity assay was used to measure the number of nonviable cells after incubation with HstN 10 µM or HstP 10 µM. PC and EtOH served as controls. The measurement was performed using CytoTox-ONE™ Homogeneous Membrane Integrity Assay (Promega, Madison, WI, USA) according to the manufacturer’s instructions.

### 2.5. ATP Measurement

A bioluminescence assay was used to measure ATP levels, which is based on the production of light from ATP and luciferin in the presence of luciferase. The measurement was performed using a ViaLight^TM^ Plus Kit (Lonza, Basel, Switzerland) according to a previously published protocol [38]. Cells incubated with DMEM served as a control.

### 2.6. Cellular Respiration

Respiration in SH-SY5Y cells was assessed with an Oxygraph-2k (Oroboros, Innsbruck, Austria) and DatLab 7.0.0.2. The cells were treated according to a complex protocol developed by Dr. Erich Gnaiger [39]. Cells were incubated with different substrates, inhibitors, and uncouplers. First, cells were washed with PBS (containing potassium chloride 26.6 mM, potassium phosphate monobasic 14.705 mM, sodium chloride 1379.31 mM, and sodium phosphate dibasic 80.59 mM) and scraped into mitochondrial respiration medium (MiRO5) developed by Oroboros [39]. Afterwards, they were centrifuged, resuspended in MiRO5, and diluted to 10^6^ cells/mL. After 2 mL of cell suspension was added to each chamber and endogenous respiration was stabilized, the cells were treated with digitonin (10 µg/10^6^ cells) to permeabilize the membrane, leaving the outer and inner mitochondrial membrane intact. OXPHOS was measured by adding complex I and II the substrates malate (2 mM), glutamate (10 mM), and ADP (2 mM), followed by succinate (10 mM). Stepwise addition of carbonyl cyanide-4-(trifluoromethoxy) phenylhydrazone showed the maximum capacity of the electron transfer system. To measure complex II activity, rotenone (0.1 mM), a complex I inhibitor, was injected. After that, oligomycin (2 µg/mL) was added to measure the leak respiration. Inhibition of complex III by the addition of antimycin A (2.5 µM) determined residual oxygen consumption, which was subtracted from all respiratory states. The activity of complex IV was measured by adding *N,N,N′,N′*-tetramethyl-*p*-phenylenediamine (0.5 mM) and ascorbate (2 mM). To measure the sodium autoxidation rate, azide (≥100 mM) was added. Afterwards, complex IV respiration was corrected for autoxidation. PC was used as control for HstN and ethanol for HstP.

### 2.7. Citrate Synthase Activity

A subsample of cell suspension from the respiratory measurement was immediately frozen in liquid nitrogen and stored at –80 °C. It was then measured according to a protocol described in a previous study [38]. PC was used as control for HstN and ethanol for HstP.

### 2.8. Real-Time qRT-PCR

Total RNA was isolated using the RNeasy Mini Kit (Qiagen, Hilden, Germany) according to the manufacturer’s guidelines. RNA was quantified using a Nanodrop^TM^ 2000c spectrometer (Thermo Fisher Scientific, Waltham, MA, USA). To remove residual genomic DNA, samples were treated with a TURBO DNA-free^TM^ kit according to the manufacturer’s instructions (Thermo Fisher Scientific, Waltham, MA, USA). Complementary DNA was synthesized from 1 µg total RNA using an iScript cDNA Synthesis Kit (Bio-Rad, Munich, Germany). qRT-PCR was conducted using a CfX 96 Connect™ system (Bio-Rad, Munich, Germany). Primers were provided from Biomol (Hamburg, Germany). All primers are listed in Table 1. The cDNA aliquots were diluted 1:10 with RNase-free water (Qiagen, Hilden, Germany), and all samples were analyzed in triplicate. PCR cycling conditions were as follows: initial denaturation for 3 min at 95 °C, followed by 45 cycles at 95 °C for 10 s, 58 °C for 30 s (or 56 °C for 45 s, depending on the primer), and 72 °C for 29 s. Expression was analyzed with −(2∆∆Cq) using Bio-Rad CfX manager software. Normalization factor was calculated based on the geometric mean of the levels of multiple control genes of *ß-actin (ACTB), glyceraldehyde-3-phosphate dehydrogenase (GAPDH)*, and *phosphoglycerate kinase 1 (PGK1)* according to the MIQE guidelines [40]. No-template control served as an assay control to exclude impurities.

### 2.9. Western Blotting

Samples (10 µg protein) were mixed with 20 mM Tris(hydroxymethyl)aminomethane buffer (pH 7.4), Laemmli Sample Buffer 2x (Bio-Rad, Hercules, CA, USA), and β-mercaptoethanol. After denaturation for 5 min at 95 °C, the samples were loaded on a Mini-PROTEAN TGX Gel 12% (Bio-Rad, Hercules, CA, USA) and separated by electrophoresis (35 min at 200 V). Gels were transferred onto a polyvinylidene fluoride (PVDF) membrane (90 min at 30 V). After incubation with blocking solution for 30 min, the membrane was washed 3 times with Tris-buffered saline containing Tween^®^20 (TBST) and incubated with primary antibodies overnight at 4 °C with constant shaking. After washing with TBST 3 times, the membrane was incubated with horseradish peroxidase (HRP)-conjugated secondary antibody (Calbiochem via Merck Millipore, Darmstadt, Germany) for 10 min at 20 °C with constant shaking. After incubation, the membrane was again washed 3 times with TBST. Visualization was carried out with Luminata™ Western HRP Substrate (Merck Millipore, Darmstadt, Germany). GAPDH served as loading control. Band analysis was performed using a ChemiDoc XRS system (Bio-Rad, Munich, Germany). Detection of respiratory system complexes was carried out by MitoProfile^®^ Total OXPHOS Rodent WB Antibody Cocktail (ab110413; Abcam, Cambridge, UK). Anti-glyceraldehyde 3-phosphate dehydrogenase (GAPDH) antibody (MAB374; Merck Millipore, Darmstadt, Germany) was used to verify equal protein loading.

### 2.10. Aβ1-40 Measurement

After 24 h incubation, the medium in the cell culture flasks was collected and the cells were rinsed with cold PBS. The suspension was then centrifuged at 220× *g* for 5 min. Then, the supernatant was discarded and the cell pellet was resuspended in 1.5 mL PBS and protease inhibitor cocktail (Merck, Darmstadt, Germany). The suspension was then centrifuged at 112× *g* for 5 min and the supernatant was removed. The cell pellet was collected and 600 µL of cell extraction buffer (Thermo Fisher Scientific, Waltham, MA, USA) was added and incubated for 30 min. After that, the suspension was centrifuged at 13,000× *g* for 10 min. The supernatant was transferred to a new tube and stored at −80 °C. The supernatant was thawed on ice and pipetted onto a 384-well plate (Greiner Bio-One, Kremsmuenster, Austria). To measure the amyloid β-protein concentration, an HTRF-Amyloid-Beta 1-40 Kit (Cisbio, Codolet, France) was used, with samples treated according to the manufacturer’s instructions. The optical density was then measured at 665 and 622 nm emission wavelengths. Aβ levels were normalized against protein content.

### 2.11. Protein Quantification

Protein content was determined using a Pierce^TM^ Protein Assay Kit (Thermo Fisher Scientific, Waltham, MA, USA) according to the manufacturer’s instructions. Bovine serum albumin was used as a standard.

### 2.12. Peroxidase Activity

Peroxidase activation was measured using an Amplex^TM^ Red Peroxidase Kit (Thermo Fisher Scientific, Waltham, MA, USA) according to the manufacturer’s instructions. Cells were seeded in 96-well plates and incubated for 24 h.

### 2.13. Peroxidase Activity of SH-SY5Y-MOCK Cells in Presence of Aβ

Aβ_25-35_ powder was dissolved and sonicated for 10 min in 5 M Tris-HCl buffer (pH 7.4) at 4 °C and aggregated at 37 °C for 16 h. Then SH-SY5Y-MOCK cells were incubated for 24 h with 10 µM and 1 µM Aβ. Peroxidase activation was measured using the Amplex^TM^ Red Peroxidase Kit (Thermo Fisher Scientific, Waltham, MA, USA) according to the manufacturer’s instructions. Tris-HCl served as control.

### 2.14. Peroxidase Activity of Cytochrome c

To determine the peroxidase activity of cytochrome c, we used 2 µM cytochrome c from horse heart (Sigma-Aldrich, St. Louis, MO, USA) dissolved in Na_2_HPO_4_ buffer (100 mM, pH 7) combined with 25 mM guaiacol (Sigma-Aldrich, St. Louis, MO, USA). Then, 10 µM of HstN or HstP was applied, and the reaction was started by adding 10 mM H_2_O_2_ (Merck, Billerica, MA, USA). The reaction temperature was set to 25 °C. The resulting orange colored product was measured at 470 nm.

### 2.15. ROS Measurement

Cellular ROS production was determined using a DCFDA/H2DCFDA Cellular ROS Assay Kit (ab113851; Abcam, Cambridge, UK). Cells were incubated for 24 h with our substances, then the manufacturer’s instructions were followed.

### 2.16. Statistics

Statistical analyses were performed by applying one-way ANOVA with Tukey´s multiple comparison post hoc test and Student’s unpaired t-test (GraphPad Prism 9 software, San Diego, CA, USA). Statistical significance was defined for *p* values as follows: ns, not significant, * *p* < 0.05, ** *p* < 0.01, *** *p* < 0.001, and **** *p* < 0.0001.

## 3. Results

### 3.1. Mitochondrial Dysfunction in SH-SY5Y-APP_695_ Cells

**Figure 1:**
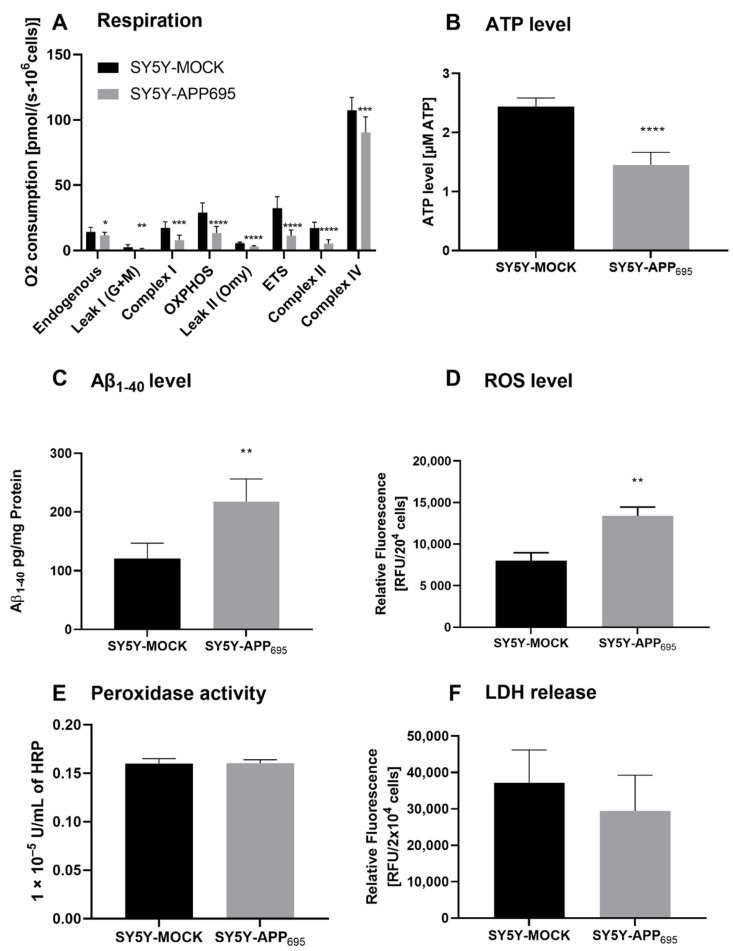
Respiration, ATP, Aβ_1-40_ level, ROS level, and peroxidase activity of SH-SY5Y-APP_695_ cells compared to SH-SY5Y-MOCK cells. (**A**) Respiration of SH-SY5Y-MOCK and -APP cells adjusted to cell count. Respective mean values ± SD are shown. *p < 0.05, **p < 0.01, ***p < 0.001, and **** *p* < 0.0001. Significance was determined by Student’s unpaired t-test, N = 12. (**B**) SH-SY5Y-APP_695_ cells exhibit reduced ATP levels compared to SH-SY5Y-MOCk control cells. ATP levels were determined after 24 h seeding by bioluminescence assay. Respective mean values ± SD are shown. Significance was determined by Student´s unpaired t-test (****p < 0.0001), N = 8. (**C**) Concentration of Aβ_1-40_ levels in SH-SY5Y-APP_695_ cells compared to SH-SY5Y-MOCK cells. ** *p* < 0.01. Significance was determined by Student’s unpaired t-test. N = 6. (**D**) ROS levels in RUF/20^4^ cells in SH-SY5Y-APP_695_ cells compared to SH-SY5Y-MOCK cells. Respective mean values ± SD are shown. Significance was determined by Student´s unpaired t-test (*** *p* < 0.001), N = 8. (**E**) Peroxidase activity in SH-SY5Y-MOCK cells compared to -APP_696_ cells. Respective mean values ± SD are shown. Significance was determined by Student´s unpaired t-test, N = 8. (**F**) Membrane integrity by LDH release level in RUF/20^4^ cells in SH-SY5Y-APP_695_ cells compared to SH-SY5Y-MOCK cells. Respective mean values ± SD are shown, N = 4.

SH-SY5Y-APP_695_ cells are an established model for the initial phase of AD [34,35]. Here, we confirm that SH-SY5Y-APP_695_ cells are deficient with regard to ATP levels and respiration but show an increase in Aβ levels compared to SH-SY5Y-MOCK cells. A significant reduction in respiration was measured across all complexes in SH-SY5Y-APP_695_ cells (Figure 1A). The significant lower respiration led to reduced ATP production in SH-SY5Y-APP_695_ cells compared with SH-SY5Y-MOCK cells (Figure 1B). In comparison, SH-SY5Y-APP_695_ cells produced significantly higher Aβ levels than SH-SY5Y-MOCK cells (Figure 1C). Figure 1D shows that the ROS levels in SH-SY5Y-APP_695_ cells were significantly higher than in SH-SY5Y-MOCK cells. There was no significant difference in peroxidase activity between the two models, as shown in Figure 1E. Figure 1F shows LDH release from SH-SY5Y-APP_695_ cells compared to SH-SY5Y–MOCK cells. There were no significant differences in the membrane integrity between the two cell lines.

### 3.2. Effect of HstN and HstP on Membrane Integrity

**Figure 2:**
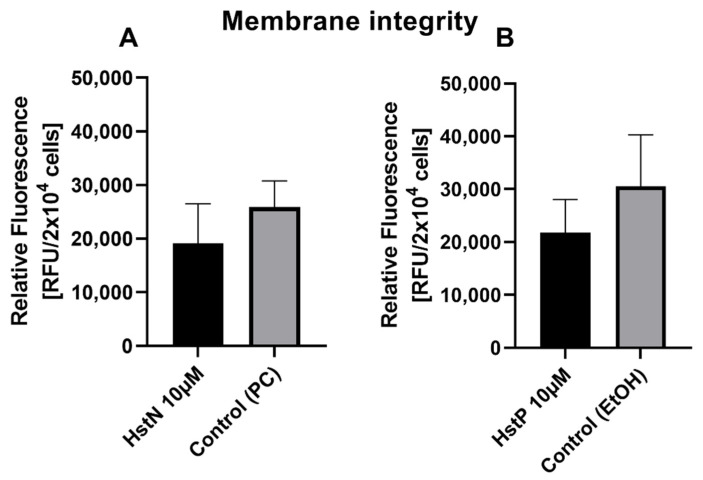
Effect of HstN and HstP on membrane integrity of SH-SY5Y-APP_695_ cells. (**A**) Effect on membrane integrity after incubation with HstN. PC served as control. (**B**) Effect on membrane integrity after incubation with HstP. EtOH served as control. Respective mean values ± SD are shown, N = 4. Significance was determined by unpaired Student’s t-test. HstN, hesperetin nanocrystal; HstP, hesperetin in pure form; PC, PlantaCare; EtOH, ethanol.

To examine the influence of HstN and HstP on cell viability, a membrane integrity assay was performed. Both substances, HstN (Figure 2A) and HstP (Figure 2B), had lowering effects but no significant effect on LDH release in SH-SY5Y-APP_695_ cells compared to the control.

### 3.3. Effect of Hesperetin and Its Nanocrystals on Mitochondrial Functions

To investigate the effect of Hst, we incubated SH-SY5Y-APP_695_ and -MOCK cells with HstN and HstP. First, we measured the effects of HstN and HstP (10 µM) on O_2_ consumption in the two types of cells after incubation for 24 h. Then, citrate synthase activity was measured as an established mitochondrial mass marker [41], and we evaluated the impact of different concentrations of HstN and HstP on ATP levels in SH-SY5Y-APP_695_ and -MOCK control cells.

**Figure 3:**
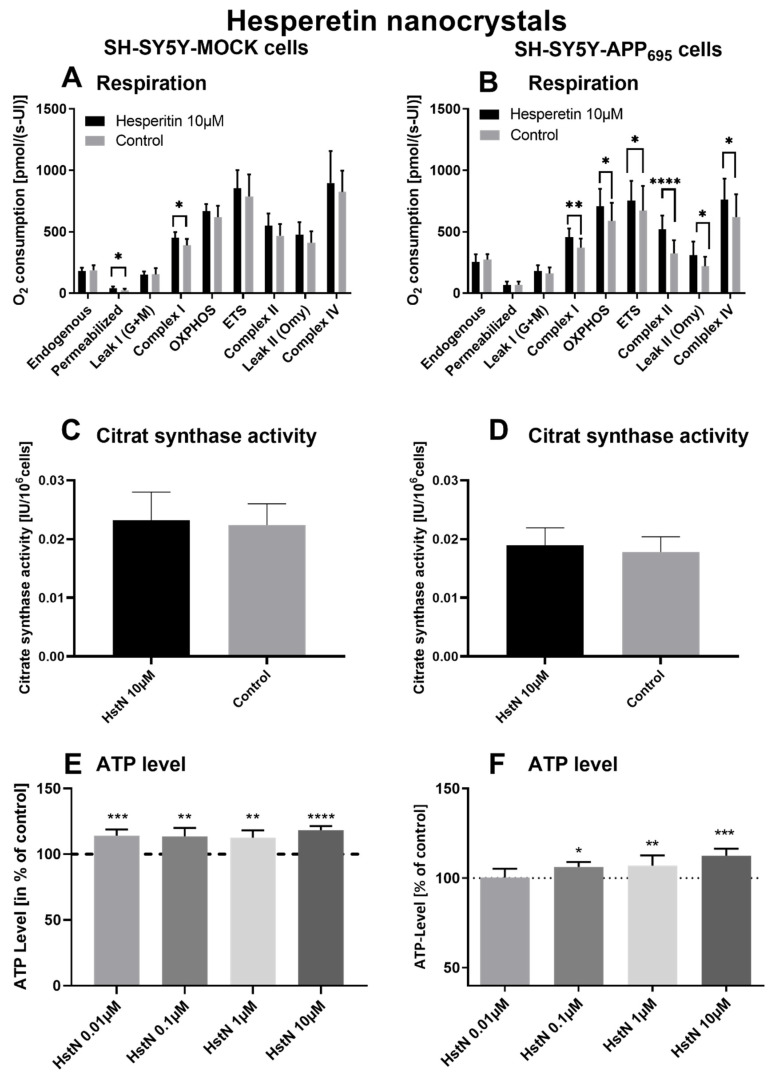
Respiration, citrate synthase activity, and ATP level of SH-SY5Y cells after incubation with hesperitin nanocrystals (HstN). Cells were incubated for 24 h with 10 µM HstN or PlantaCare as control, N = 12. Respective mean values ± SD are shown. * *p* < 0.05, ** *p* < 0.01, *** *p* < 0.001 and **** *p* < 0.0001. Significance was determined by Student’s unpaired t-test. Respiration of (**A**) SH-SY5Y-MOCK and (**B**) SH-SY5Y-APP_695_ cells adjusted to international units (IU) of citrate synthase activity. Citrate synthase activity of (**C**) SH-SY5Y-MOCK cells and (**D**) SH-SY5Y-APP_695_ cells. Values are given as IU of citrate synthase activity. ATP level of (**E**) SH-SY5Y-MOCK cells and (**F**) SH-SY5Y-APP_695_ cells after 24 h incubation with 0.01–10 µM HstN. Cells treated with cell culture medium served as control (100%).

Incubation of HstN had a significant increasing effect on complex I (*p* = 0.0155) of the O_2_ flux in SH-SY5Y-MOCK cells adjusted to the cell count (Figure 3A). HstN had a significant effect of increasing the activity of all complexes of the respiratory chain (Figure 3B). The activity of complex I (*p* = 0.053), OXPHOS (complex I + II; *p* = 0.0241), and complex IV (*p* = 0.0468) as well as ETC (*p* = 0.0162) and leak II respiration (*p* = 0.0168) were significantly enhanced. Complex II showed the greatest changes compared to the control (*p* < 0.0001) when O_2_ flux was adjusted to the citrate synthase activity (CS). To establish whether the increased activity was a result of an enhanced mitochondrial mass or higher activity of the complexes, CS was measured. CS represents an established marker for mitochondrial mass [42]. We found no significant effects between the incubation of HstN and control in both cell lines, as shown in Figure 3C,D.

To test the effect of HstN on ATP levels, SH-SY5Y-MOCK and -APP_695_ cells were incubated with different concentrations for 24 h (Figure 3E,F). The 10 µM HstN concentration had the greatest influence (*p* < 0.0001). SH-SY5Y-MOCK cells showed increased levels of ATP after incubation with HstN compared to the control. On average, the ATP levels of cells incubated with HstN were 15% higher than those of the control group. SH-SY5Y-APP_695_ cells showed increased ATP levels after incubation with 0.01 to 10 µM HstN. The 10 µM concentration had the greatest impact (*p* = 0.008). On average, the ATP levels were 6% higher compared to controls.

In the next step, we examined the effect of HstP on the complex activity of the respiratory chain (Table 2). It turned out that HstP had no significant effect on SH-SY5Y-MOCK and -APP_695_ cells. To investigate whether HstP had an effect on the mitochondrial mass, we measured the citrate synthase activity. There were no significant differences between HstP and the solvent group with regard to the citrate synthase activity in both SH-SY5Y cell lines.

To investigate the effects of HstP on the ATP level, SH-SY5Y-MOCK cells were incubated for 24 h with different concentrations of HstP (Table 2). Significant elevating effects were observed with an HstP concentration of 1 µM, with ATP levels on average 9% higher than in the control group.

3.4. qRT-PCR and Western Blot Analysis

**Figure 4:**
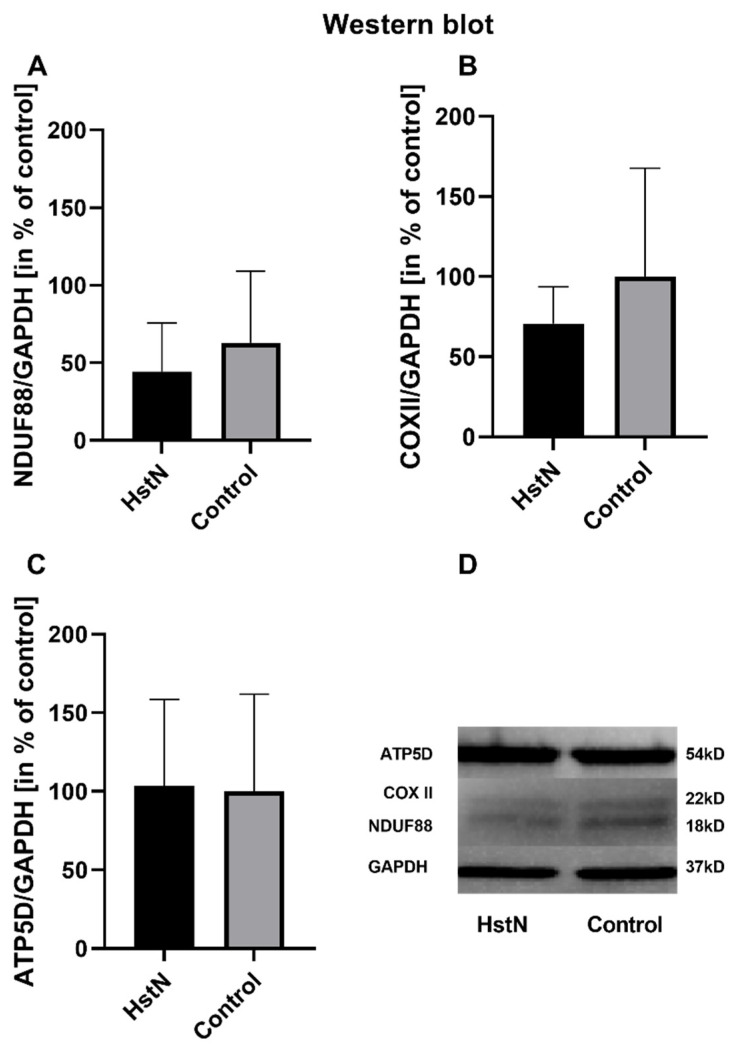
Western blot analysis of mitochondrial respiratory chain complexes of five experiments. Protein levels of (**A**) complex I subunit *NDUF88*, (**B**) complex IV subunit *COXII*, and (**C**) complex V subunit *ATP5D* in % of control after incubation with 10 µM HstN or PC (control) in SH-SY5Y-APP_695_ cells. (**D**) Lower part: representative Western blots of one experiment. GAPDH served as loading control. Respective mean values ± SD are shown. Significance was determined by Student’s unpaired t-test. HstN, hesperetin nanocrystal.

The results of the relative mRNA expression are shown in Table 3. HstN had no effect on the relative mRNA expression of citrate synthase (*CS*), *COX subunit 5A* (*COX5A*), *NADH-dehydrogenase flavoprotein 1* (*CI*), or *ATP-synthase delta-subunit* (*ATP5D*) in SH-SY5Y-MOCK cells. On the other hand, HstN had a significant boost effect on the expression of genes in SH-SY5Y-APP_695_ cells. Expression of the complex I gene showed significantly increased values between HstN and PC (*p* = 0.0012). There were also significant elevated mRNA values of *COX5A* (*p* = 0.0275) and *ATP5D* (*p* = 0.0229). HstN-related increased gene expression did not result in enhanced protein levels of subunit *NDUF88* (complex I), *COX-II* (complex IV), or *ATP5D* (complex V) (Figure 4).

### 3.5. Production of Aβ_1-40_

**Figure 5:**
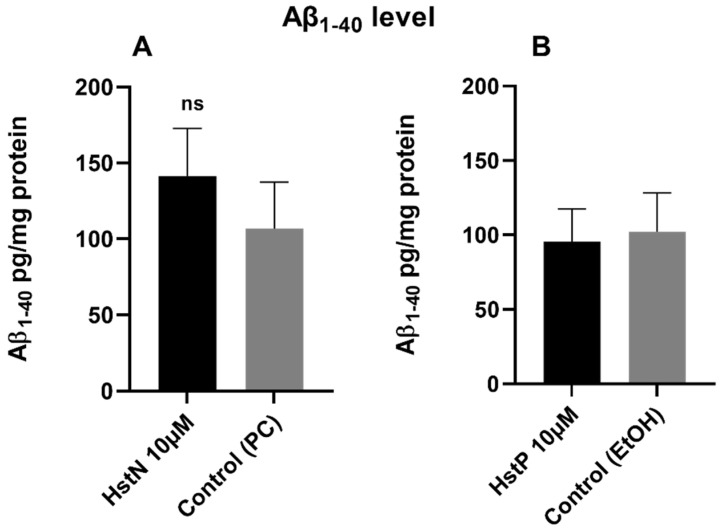
Effect of HstN and HstP on Aβ1-40 levels in SH-SY5Y-APP_695_ cells. Concentration of Aβ_1-40_ in SH-SY5Y-APP_695_ cells: (**A**) incubated for 24 h with HstN, with PC as control, and (**B**) incubated for 24 h with HstP, with ethanol as control. Values are adjusted to protein concentrations. Respective mean values ± SD are shown, N = 6. Significance was determined with unpaired Student’s t-test. ns, not significant; HstN, hesperetin nanocrystal; HstP, hesperetin in pure form; PC, PlantaCare; EtOH, ethanol.

To investigate whether HstN or HstP had an effect on Aβ_1-40_ levels, SH-SY5Y-APP_695_ cells were incubated for 24 h with the Hst preparations or solvent control PC or EtOH. The results in Figure 5 show that HstN and HstP had no significant effect on Aβ_1-40_ levels compared to their respective solvent control in SH-SY5Y-APP_695_ cells. However, is obvious that incubation with HstN increased the expression of Aβ_1-40_ numerically by about 50% (Figure 5A).

### 3.6. Peroxidase Activity

**Figure 6:**
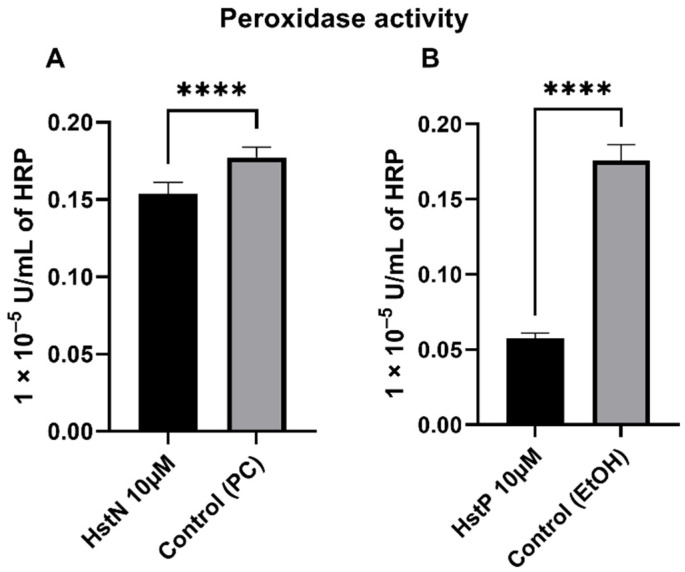
Effect of HstN and HstP on peroxidase activity in SH-SY5Y-APP_695_ cells. Peroxidase activity in SH-SY5Y-APP_695_ cells: (**A**) incubated for 24 h with HstN, with PC as control, and (**B**) incubated for 24 h with HstP, with ethanol as control. Respective mean values ± SD are shown, N = 10. Significance was determined by unpaired Student’s t-test. **** *p* < 0.0001. HstN, hesperetin nanocrystal; HstP, hesperetin in pure form; PC, PlantaCare; EtOH, ethanol.

In order to investigate the effects of HstN and HstP on peroxidase activity, SH-SY5Y-APP_695_ cells were incubated for 24 h with the appropriate substances. HstN (Figure 6A) had a significant lowering effect on peroxidase activity compared to the control (*p* < 0.0001). HstP (Figure 6B) had an even stronger lowering influence on peroxidase activity compared to the control (*p* < 0.0001).

### 3.7. Peroxidase Activity of SH-SY5Y-MOCK Cells Incubated with Aβ

**Figure 7:**
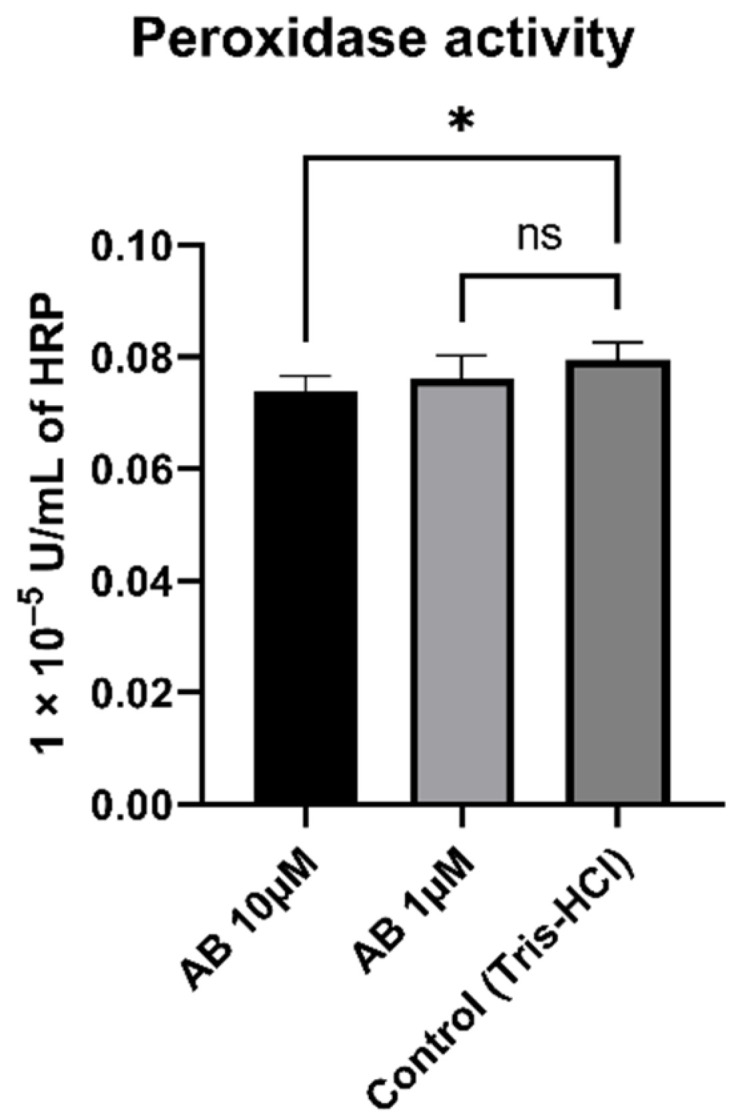
Effect of Aβ on peroxidase activity in SH-SY5Y-MOCK cells incubated with different concentrations of Aβ for 24 h. Respective mean values ± SD are shown, N = 7. Significance was determined by one-way ANOVA. * *p* < 0.05. Aβ, amyloid beta.

To determine whether peroxidase activity is a result of Aβ, we incubated SH-SY5Y-MOCK cells with different concentrations of aggregated Aβs (Figure 7). The 10 µM Aβ concentration had a significant (*p* = 0.012) lowering effect on the peroxidase activity in comparison to the control. The 1 µM concentration had no significant effect on peroxidase activity in SH-SY5Y-MOCK cells.

### 3.8. Peroxidase Activity of Cytochrome c

**Figure 8:**
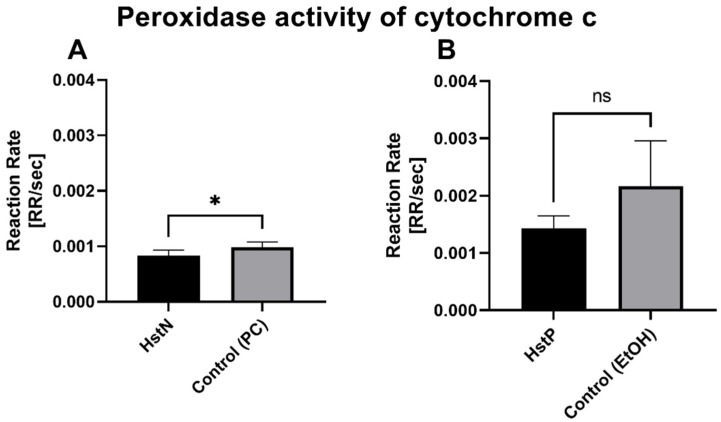
Effects of HstN and HstP on peroxidase activity of cytochrome c: (**A**) incubated with HstN, with PC as control (N = 6), and (**B**) incubated with HstP, with ethanol as control (N = 5). Respective mean values ± SD are shown. Significance was determined with unpaired Student’s t-test. * *p* < 0.05. HstN, hesperetin nanocrystal; HstP, hesperetin in pure form; PC, PlantaCare; EtOH, ethanol.

To test whether the lower peroxidase activity was a result of reduced activity of cytochrome c, which can act in a similar way to a peroxidase, we measured the activity of cytochrome c in the presence of HstN (Figure 8A) and HstP (Figure 8B). The results show that HstN significantly decreased the activity of cytochrome c as a peroxidase compared to the control (*p* = 0.0225). HstP had a lowering effect but not a significant one.

### 3.9. ROS Measurement

**Figure 9:**
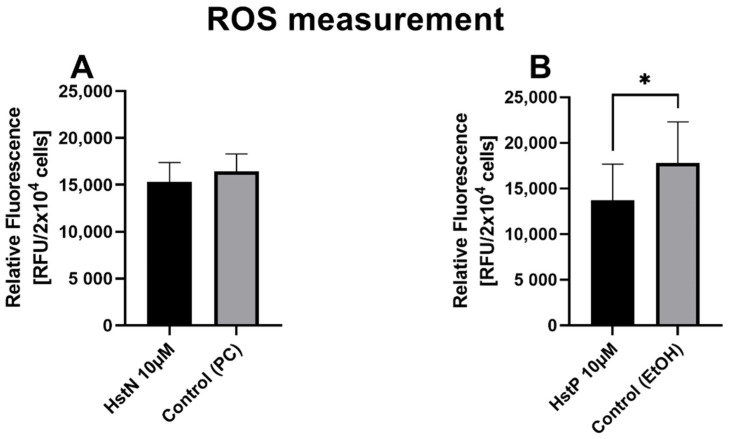
Effect of HstN and HstP on expression of ROS production in SH-SY5Y-APP_695_ cells: (**A**) incubated for 24 h with HstN, with PC as control, and (**B**) incubated for 24 h with HstP, with ethanol as control. Respective mean values ± SD are shown, N = 10. Significance was determined with unpaired Student’s t-test. * *p* < 0.05. HstN, hesperetin nanocrystal; HstP, hesperetin in pure form; PC, PlantaCare; EtOH, ethanol.

In the next step, we examined the effects of HstN and HstP on general ROS expression in SH-SY5Y-APP_695_ cells. We found a small lowering effect of ROS after incubation with HstN (Figure 9A). In contrast, HstP (Figure 9B) had a significant decreasing effect on ROS production compared to the control (*p* = 0.045).

## 4. Discussion

In the present work, we examined the effects of HstN and HstP in a cellular model of early Alzheimer’s disease, focusing on mitochondrial function in SH-SY5Y cells. Mitochondrial dysfunction is an early indicator of AD [10].

### Effect of Hesperetin on Mitochondrial Function in SH-SY5Y-MOCK and -APP_695_ Cells

Mitochondria are the powerhouses of cells and responsible for energy production in the form of ATP. To investigate the effects on mitochondrial function, we incubated SH-SY5Y-MOCK cells with HstN and measured the ATP levels (Figure 3E). All concentrations of HstN significantly enhanced ATP levels. The 10 µM concentration had the greatest effect. Biesemann et al., who incubated human muscle cells with HstP, reported similar results. They pointed out that 10 µM HstP had the highest effect on increasing cellular ATP levels [43]. Mitochondria are not only the main producers but also targets of ROS [44,45]. Mitochondrial ROS production has a negative effect on ATP levels [45]. A possible explanation for the effect of HstP is that this flavonoid [46] has scavenging effects and protects the cells against ROS-induced damage. Previous work showed that HstP functions as an antioxidant and protects cells against oxidative stress [29,47]. This effect could lead to increased production of ATP compared to untreated controls. Dissolving compounds in solvents is commonly performed when testing them in cell cultures; however, harmful effects such as precipitation, induced oxidative stress and complications must also be considered when applying them in vivo. To circumvent this, we developed a nano-formulation of Hst and compared the effects with its pure form (dissolved). SH-SY5Y-MOCK and -APP_695_ cells were incubated with different concentrations of HstP. Regarding ATP levels, only the 1 µM concentration of HstP had a significant enhancing effect in both cell types (Table 2). A possible explanation could be the particle size of the nanocrystals. In a previous study, we determined that Hst with smaller particles had a greater effect on elevating ATP levels in SH-SY5Y-APP_wt_ cells compared to larger ones [25]. Similar, most studies have shown that the particle size has an impact on cell functions [48,49,50]. Thus, HstP may have had a lower effect on ATP levels due to its particle size.

To investigate whether HstN or HstP would influence cell membrane integrity, we incubated SH-SY5Y-APP_695_ cells with both substances (Figure 2). LDH release is a good marker for cell viability [51], and here, HstN and HstP seemed to improve cell viability and lower LDH release in the surrounding medium but not significantly.

To test the impact of Hst on O_2_ flux, we incubated SH-SY5Y-MOCK and -APP_695_ cells with HstN and HstP. Incubation of both types of cells with HstP did not significantly alter O_2_ consumption (Table 2). Moreover, it did not affect citrate synthase activity, which is a good biomarker for mitochondrial mass [42].

When SH-SY5Y-MOCK cells were incubated with HstN and adjusted to citrate synthase activity, complex I showed significant improvement O_2_ consumption compared to the control (Figure 3A). As complex I activity plays an integral part in creating the proton gradient necessary for energy production, a significant increase in its activity is reflected in increased ATP levels (Figure 3E). The increased complex activity cannot be explained by increased mitochondrial mass, as citrate synthase activity was unchanged (Figure 3C). In SH-SY5Y-APP_695_ cells incubated with HstN, all complexes showed increased activity compared to controls (Figure 3B). To study whether the increased complex activity was a result of increased mitochondrial mass, we measured the citrate synthase activity, which again showed no difference between the two groups (Figure 3D). The Aβ deposition resulting from AD could have a negative effect on the electron transport chain, most notably on complex I and IV [52,53]. This leads to lower activity of the respiratory chain and reduced energy metabolism [54]. Damaged or inhibited respiratory chain complexes produce more ROS, leading to increased oxidative stress [55]. These effects seem to be mitigated by HstN, so the incubated groups had higher complex activity. Kheradmand et al. and Moghaddam et al. showed that HstP and HstN upregulate antioxidative mechanisms; for example, catalase (CAT), superoxide dismutase (SOD), glutathione reductase (GRx), and glutathione (GSH) levels were upregulated in a rat model of AD [31,56]. Thus, HstN has a greater effect compared to HstP. Due to the activation of those mechanisms, the antioxidative effect of Hst may not only be a result of scavenging ROS but also be mediated by the activation of antioxidative systems, which, in turn, would lead to enhanced complex activity compared to control cells.

Furthermore, we studied the effects of HstN on the expression of genes encoding the production of key protein complexes of the respiratory chain. The expressions of *CS*, *COX5A*, *CI*, and *ATP5D* were determined after incubation of SH-SY5Y-MOCK and -APP_695_ cells with HstN (Table 3). HstN had no significant effect on the mRNA levels in SH-SY5Y-MOCK cells. Incubation of SH-SY5Y-APP_695_ cells with Hst significantly increased the expression of *CI*. HstN also significantly upregulated the gene expression of *COX5A* and *ATP5D*. Mastroeni et al. reported that the gene expressions of complexes I, IV, and V were reduced in patients with AD [57]. Biesemann et al. observed that 10 µM of HstP increased the expressions of complexes I, III, and IV in human muscle cells [43]. Similarly, in our study, HstN increased the mRNA levels of all three investigated complexes, suggesting increased production of the complexes. However, despite elevated mRNA levels, no increased complex protein levels were found (Figure 4). It has been shown that elevated gene expression is not necessarily associated with increased protein production [58]. Thus, the increased complex activity and the resulting elevated ATP levels in SH-SY5Y-APP_695_ cells after HstN incubation might not be explained by higher amounts of protein.

Next, we examined the effects of Hst on the production of Aβ in SH-SY5Y-APP_695_ cells (Figure 5). Hst in its pure and nano forms had no significant impact on Aβ1-40 levels. Thus, the advantageous effects of HstN on mitochondrial function might be independent of the Aβ pathway. Similarly, we recently reported that olesoxime, a modulator of the mitochondrial permeability transition pore, improved mitochondrial dysfunction and increased Aβ levels in Thy1-AβPP_SL_ mice and HEK_293_ cells [59]. As Hst also interacts with membranes [60,61], future studies should explore whether this interaction affects APP processing at the plasma membrane [62].

Then, we investigated the effects of HstN and HstP on peroxidase activity. Both substances had a lowering effect on peroxidase activity (Figure 6). We hypothesize that the lower peroxidase activity is a result of reduced peroxidase activity of cytochrome c. Cytochrome c can act in a similar way to a peroxidase, which will then catalyst the oxidation of cardiolipin. This promotes an early stage of apoptosis and the release of cytochrome c, which will accelerate cell death [63,64,65]. HstN had a significant reducing effect on cytochrome c activity (Figure 8A). HstP seemed to reduce the activity, but this was not significant (Figure 8B). Potentially higher peroxidase activity may be a result of complex formation from Aβ with heme, which can act in a similar way to a peroxidase [66,67,68]. To investigate this, we incubated SH-SY5Y-MOCK cells with low levels of Aβ with different concentrations of aggregated Aβ (Figure 7). Contrary to our original assumption, Aβ actually decreased peroxidase activity instead of increasing it. A possible explanation could be the aggregation condition of the Aβ peptides. Yuan et al. found that non-aggregated Aβ had higher peroxidase activity compared with aggregated [67]. A second possible explanation could be the Aβ peptide used. Here, we used Aβ_25-35_, which is more neurotoxic than Aβ_1-40_ or Aβ_1-42_ [69] but maybe does not bind as well as those other peptides to heme to increase peroxidase activity [67].

In the last step, we measured the ROS level in SH-SY5Y-APP695 cells (Figure 9) to confirm our hypothesis that Hst had a protective effect against ROS, which would explain the increased ATP levels and complex activity. We found that HstP had a significantly greater effect on reducing ROS compared to HstN. This contradicts our theory that the increased ATP levels and complex activity were due to increased protection by ROS. Thus, the effects shown are not solely due to the protective effect against ROS in HstN.

We conclude that HstN has a larger influence on mitochondrial functions, especially in the SH-SY5Y-APP_695_ AD cell model. Similarly, Kheradmand et al. showed greater effects for HstN in a Wistar AD rat model [31].

At this point, it should be noted that the cell model used, which was described as a model of early Alzheimer’s disease, is essentially an Aβ overexpression model that does not, of course, reflect the complex pathophysiological processes of neurodegeneration. Nevertheless, our data may help to understand the molecular basis of the effects of Hst on the disease process.

## 5. Conclusions

In this study, we investigated the effects of Hst in its pure and nanocrystal forms on mitochondrial functions in SH-SY5Y-APP_695_ cells. Hst nanocrystals had a superior beneficial impact on mitochondrial dysfunction compared to the pure form in a cellular model of early AD. The beneficial results in mitochondrial function may be linked to effects on gene expression but not on the expression of complex protein levels, including Aβ production. Furthermore, HstN and HstP reduce peroxidase activity, and especially HstN reduces the activity of cytochrome c as a peroxidase.

## Figures and Tables

**Table 1:** Oligonucleotide primer sequences, product sizes, and primer concentrations for quantitative real-time PCR.

Primer	Sequence	Manufacturer	Product Size	Concentration (nM)
*ß-Actin (* *ACTB)* *NM_001101.2*	5′-ggacttcgagcaagagatgg-3′ 5′-agcactgtgttggcgtacag-3′	Biomol, Hamburg, Germany	234	200
*ATP-synthase delta-subunit* *(* *ATP5D)* *NM_001687*	5′-ggaagctcctcctcagcttt-3′ 5′-caggcttccgggtctttaat-3′	Biomol, Hamburg, Germany	198	200
*COX subunit 5A* *(* *COX5A)* *NM_004255.3*	5′-gcatgcagacggttaaatga-3′ 5′-agttcctccggagtggagat-3′	Biomol, Hamburg, Germany	152	200
*Citrate synthase* *(* *CS)* *NM_004077*	5′-ccatccacagtgaccatgag-3′ 5-ctttgccaacttccttctgc-3′	Biomol, Hamburg, Germany	186	400
*NADH-dehydrogenase flavoprotein 1* *(CI)* *NM_007103.3*	5′-cgccacctagcgtctctatc-3′ 5′-tgaaaatccggtcttcatcc-3′	Biomol, Hamburg, Germany	213	200
*Glyceraldehyde-3-phosphate dehydrogenase* *(GAPDH)* *NM_002046.2*	5′-gagtcaacggatttggtcgt-3′ 5′-ttgattttggagggatctcg-3′	Biomol, Hamburg, Germany	238	200
*Phosphoglycerate kinase 1* *(PGK1)* *NM_000291.2*	5′-ctgtgggggtatttgaatgg-3′ 5′-cttccaggagctccaaa-3′	Biomol, Hamburg, Germany	198	200

**Table 2:** Respiration, citrate synthase activity, and ATP levels of SH-SY5Y cells after incubation with HstP and HstN. For respiration, cells are incubated for 24 h with 10 µM HstP and HstN or EtOH and PC as control, N = 10 for HstP and N = 12 for HstN. Respective mean values are shown. * *p* < 0.05, ** *p* < 0.01, *** *p* < 0.001 and **** *p* < 0.0001 compared to control. Significance was determined with Student’s unpaired t-test. Respiration of SH-SY5Y-MOCK and -APP_695_ cells was adjusted to international units (IU) of citrate synthase activity. ATP levels are given in % of control and were measured after 24 h of incubation with 0.01–10 µM HstP. Cells treated with DMEM served as a control. HstP, hesperetin in pure form; HstN, hesperetin nanocrystals.

*Experiment*		*SH-SY5Y-MOCK*		*SH-SY5Y-APP_695_*		*SH-SY5Y-MOCK*		*SH-SY5Y-APP_695_*	
		HstP 10 µM	Control	HstP 10 µM	Control	HstN 10 µM	Control	HstN 10 µM	Control
*Oxygraph* *O_2_ consumption (pmol/(s-UI))*	Endogenous	318 ± 45	311 ± 77	301 ± 47	322 ± 42	180 ± 26	187 ± 40	254 ± 62	273 ± 45
	Permeabilized	18 ± 11	23 ± 20	32 ± 14	37 ± 12	40 ± 15 *	24 ± 13	65 ± 30	69 ± 30
	Leak I (G+M)	102 ± 32	116 ± 22	155 ± 25	174 ± 32	151 ± 27	153 ± 51	179 ± 48	159 ± 51
	Complex I	686 ± 96	643 ± 140	546 ± 140	547 ± 155	452 ± 45 *	389 ± 53	454 ± 73 **	370 ± 74
	OXPHOS	1185 ± 163	1091 ± 358	758 ± 286	865 ± 346	669 ± 57	619 ± 94	710 ± 140 *	588 ± 149
	ETC	1395 ± 219	1275 ± 449	874 ± 274	1064 ± 240	854 ± 148	787 ± 181	753 ± 134 *	672 ± 182
	Complex II	958 ± 112	883 ± 138	600 ± 220	739 ± 179	549 ± 100	468 ± 94	522 ± 110 ****	324 ± 107
	Leak II (Omy)	910 ± 171	850 ± 297	608 ± 242	608 ± 261	477 ± 101	410 ± 93	311 ± 110 *	219 ± 78
	Complex IV	1519 ± 190	1446 ± 232	411 ± 248	415 ± 223	898 ± 258	824 ± 173	761 ± 172 *	619 ± 186
*Citrate synthase activity (IU/10^6^ cells)*		0.01378 ± 0.02	0.0138787 ± 0.004	0.01699 ± 0.001	0.0191789 ± 0.002	0.0231924 ± 0.005	0.0223699 ± 0.004	0.018955 ± 0.003	0.0178201 ± 0.003
*ATP levels (% of control)*	0.01 µM	105 ± 11		104 ± 3		114 ± 5 ***		100 ± 5	
	0.1 µM	108 ± 6		108 ± 3		114 ± 7 **		106 ± 3 *	
	1 µM	109 ± 7 *		109 ± 2 *		113 ± 6 **		107 ± 6 **	
	10 µM	100 ± 10		107 ± 6		118 ± 3 ****		113 ± 4 ***	

**Table 3:** Effect of HstN at relative normalized mRNA expression levels in SH-SH-SY5Y-MOCK and -APP_695_ cells after incubation with HstN for 24 h, determined using quantitative real-time PCR compared to SH-SY5Y cells incubated with PlantaCare (PC). mRNA expression of control cells incubated with PC is set as 100%. Respective mean values ± SD are shown. Significance was determined with unpaired Student’s t-test (* *p* < 0.05, ** *p* < 0.01). Calculation of normalization factor based on geometric mean of multiple control genes levels of *ß-actin* (*ACTB*), *glyceraldehyde 3-phosphate dehydrogenase* (*GAPDH*), and *phosphoglycerate kinase 1* (*PGK1*). HstN, hesperetin nanocrystals.

*Complex*	SH-SY5Y-MOCK Cells	SH-SY5Y-APP_695_ Cells
*CS*	108.0 ± 12.44	121.1 ± 45.81
*CI*	66.38 ± 20.32	207.0 ± 27.35 **
*COX5A*	86.27 ± 12.89	195.6 ± 39.51 *
*ATP5D*	89.54 ± 13.9	184.5 ± 33.59 *

## Data Availability

All datas in the manuscript.
